# [Corrigendum] Compound 225# inhibits the proliferation of human colorectal cancer cells by promoting cell cycle arrest and apoptosis induction

**DOI:** 10.3892/or.2025.9017

**Published:** 2025-11-06

**Authors:** Xiaoxue Zhang, Liujun He, Yong Li, Yifei Qiu, Wujing Hu, Wanying Lu, Huihui Du, Donglin Yang

Oncol Rep 51: 70, 2024; DOI: 10.3892/or.2024.8729

Subsequently to the publication of the above paper, the authors have drawn to the attention of the Editorial Office that they made an error in assembling the western blot data in Fig. 3C on p. 6; namely, that the western blot data correctly selected for the P53 protein with the SW620 cell line (right-hand gels) had inadvertently also been included for the γ-H2A.X protein blots with the HCT116 cell line (left-hand gels). Upon analysing this figure further in the Editorial Office, we notified the authors of possibly overlapping α-tubulin control blots for the SW62 cell line in the same figure part, and the authors realized that one of these blots had similarly been chosen incorrectly.

The revised version of Fig. 3, now showing the correct γ-H2A.X data for the HCT116 cell line and the correct α-tubulin protein blots for the SW620 cell line, is shown on the next page. The authors wish to emphasize that the corrections made to this figure do not affect the overall conclusions reported in the paper, and they are grateful to the Editor of *Oncology Reports* for allowing them the opportunity to publish this corrigendum. All the authors agree with the publication of this corrigendum, and also apologize to the readership for any inconvenience caused.

## Figures and Tables

**Figure 3.**
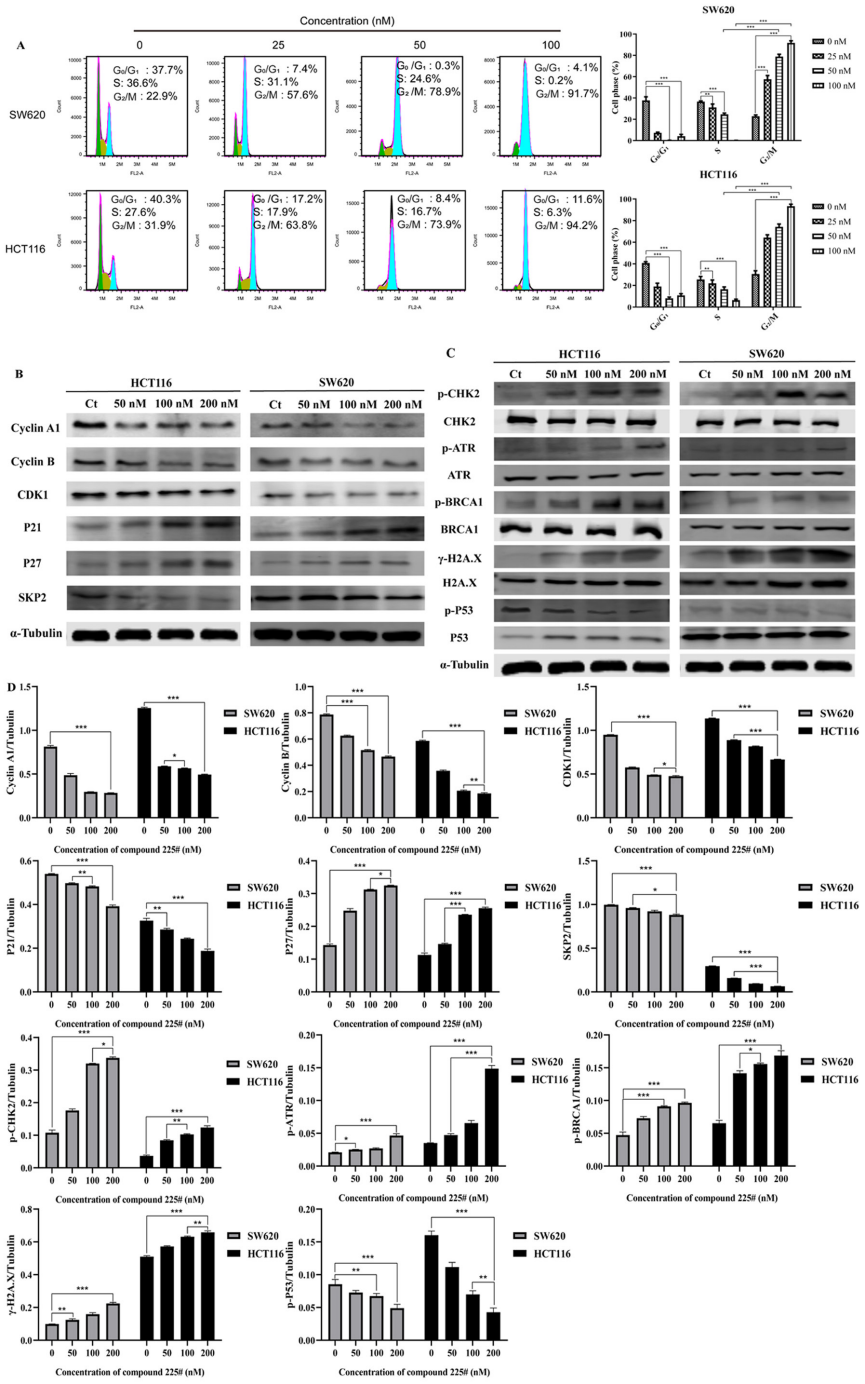
Effect of compound 225# on the apoptosis of SW620 and HCT116 cells. (A) SW620 and HCT116 cells were stained with Annexin V/PI after treatment with compound 225#. Representative flow cytometry plots are shown. (B) Effects of compound 225# on the expression levels of apoptosis-related proteins. (C) Gray values of the relevant protein bands are shown. Data are presented as the mean ± SD. *P<0.05, **P<0.01, ***P<0.001; ns, not significant; Ct, control.

